# Genetic Polymorphisms in XRCC1, CD3EAP, PPP1R13L, XPB, XPC, and XPF and the Risk of Chronic Benzene Poisoning in a Chinese Occupational Population

**DOI:** 10.1371/journal.pone.0144458

**Published:** 2015-12-17

**Authors:** Ping Xue, Lin Gao, Sha Xiao, Guopei Zhang, Mingyang Xiao, Qianye Zhang, Xiao Zheng, Yuan Cai, Cuihong Jin, Jinghua Yang, Shengwen Wu, Xiaobo Lu

**Affiliations:** 1 Department of Toxicology, School of Public Health, China Medical University, Shenyang, Liaoning, P.R. China; 2 Poisoning Department, Shenyang ninth people’s Hospital, Shenyang, Liaoning, P.R. China; Duke Cancer Institute, UNITED STATES

## Abstract

**Objectives:**

Individual variations in the capacity of DNA repair machinery to relieve benzene-induced DNA damage may be the key to developing chronic benzene poisoning (CBP), an increasingly prevalent occupational disease in China. ERCC1 (Excision repair cross complementation group 1) is located on chromosome 19q13.2–3 and participates in the crucial steps of Nucleotide Excision Repair (NER); moreover, we determined that one of its polymorphisms, ERCC1 rs11615, is a biomarker for CBP susceptibility in our previous report. Our aim is to further explore the deeper association between some genetic variations related to ERCC1 polymorphisms and CBP risk.

**Methods:**

Nine single nucleotide polymorphisms (SNPs) of XRCC1 (X-ray repair cross-complementing 1), CD3EAP (CD3e molecule, epsilon associated protein), PPP1R13L (protein phosphatase 1, regulatory subunit 13 like), XPB (Xeroderma pigmentosum group B), XPC (Xeroderma pigmentosum group C) and XPF (Xeroderma pigmentosum group F) were genotyped by the Snapshot and TaqMan-MGB^®^ probe techniques, in a study involving 102 CBP patients and 204 controls. The potential interactions between these SNPs and lifestyle factors, such as smoking and drinking, were assessed using a stratified analysis.

**Results:**

An XRCC1 allele, rs25487, was related to a higher risk of CBP (*P*<0.001) even after stratifying for potential confounders. Carriers of the TT genotype of XRCC1 rs1799782 who were alcohol drinkers (OR = 8.000; 95% CI: 1.316–48.645; *P* = 0.022), male (OR = 9.333; 95% CI: 1.593–54.672; *P* = 0.019), and had an exposure of ≤12 years (OR = 2.612; 95% CI: 1.048–6.510; *P* = 0.035) had an increased risk of CBP. However, the T allele in PPP1R13L rs1005165 (P<0.05) and the GA allele in CD3EAP rs967591 (OR = 0.162; 95% CI: 0039~0.666; *P* = 0.037) decreased the risk of CBP in men. The haplotype analysis of XRCC1 indicated that XRCC1 rs25487^A^, rs25489^G^ and rs1799782^T^ (OR = 15.469; 95% CI: 5.536–43.225; *P*<0.001) were associated with a high risk of CBP.

**Conclusions:**

The findings showed that the rs25487 and rs1799782 polymorphisms of XRCC1 may contribute to an individual’s susceptibility to CBP and may be used as valid biomarkers. Overall, the genes on chromosome 19q13.2–3 may have a special significance in the development of CBP in occupationally exposed Chinese populations.

## Introduction

Benzene, a commonly used industrial chemical, is an established human carcinogen [1]. It has been documented that long-term occupational exposure to benzene may induce chronic benzene poisoning (CBP), which could act on the bone marrow and peripheral blood cells and cause leukemia or other hematopoietic cancers in humans [2,3]. However, the susceptibility to benzene toxicity varies among individuals after a similar occupational exposure [4]. This suggests that in addition to environmental exposure, genetic polymorphisms render some individuals more susceptible to CBP. Therefore, it is critical to identify valid biomarkers in the susceptible population after occupational benzene exposure and to predict the risk of developing CBP before the disease occurs.

Benzene produces adverse effects through its metabolites, quinones and reactive oxygen species (ROS) [5–7]. Benzene’s reactive intermediates can form DNA adducts by covalently binding to macromolecules, including DNA and proteins, and ultimately give rise to single- or double-strand DNA damage [8]. The genetic damage induced by occupational exposure to benzene could directly cause oxidative damage and covalent DNA adducts [9]. Thus, a properly functioning DNA repair process is required to maintain genomic stability and prevent the mutated cells from subsequently developing into malignancies [10]. Normally, the DNA damage caused by the benzene metabolites could initiate the cellular DNA repair system. Variations in DNA repair genes may result in an individual’s susceptibility to CBP. Benzene-induced DNA damage would primarily be repaired by the NER and BER systems. BER is responsible for repairing chemically induced DNA damage at a single base, including the removal of oxidative damage and single-strand breaks. NER is involved in the restoration of a wide variety of DNA damage and structural distortions to the DNA double helix, including the large benzene-induced DNA adducts. XRCC1 (X-ray repair cross-complementing 1) acts as an indispensable factor in BER, and a clear relationship between its polymorphisms and chronic benzene poisoning has been identified. Cooperating with ADPRT (ADP ribosyltransferase), DNA polymerase β and DNA ligase III, XRCC1 could repair the gaps left acts as a scaffold after the excision of benzene DNA adducts (p-benzoquinone)[11–15]. As shown in our previous studies, a SNP at codon 118 (rs11615) of ERCC1 (Excision repair cross complementation group 1), a key element in the NER system, could modify an individual’s risk of developing CBP. We hypothesized that genetic polymorphism in other NER genes, such as XPB (Xeroderma pigmentosum group B), XPC (Xeroderma pigmentosum group C) and XPF (Xeroderma pigmentosum group F), which have not been studied, may be related to an individual’s susceptibility to benzene toxicity.

Interestingly, both XRCC1 and ERCC1 are located on chromosome 19q13.2–3, an active zone involved in DNA repair, apoptosis, and cell proliferation. PPP1R13L (protein phosphatase 1, regulatory subunit 13 like) and CD3EAP (CD3e molecule, epsilon-associated protein) are also located in this region and situated between ERCC1 and ERCC2. Previous studies have demonstrated a relationship between the risk of developing cancer and polymorphisms located in this region [16–19]. Moreover, it was reported that the overlapping genes ERCC1, CD3EAP, and PPP1R13L may interact in apoptosis and DNA repair pathways [16, 17, 20, 21]. PPP1R13L is an inhibitor of p53 that primarily affects apoptosis [22]. CD3EAP encodes a nucleoprotein that is localized to the fibrillar centers of the nucleolus and may be a member of the RNA polymerase I transcription complex. In addition, CD3EAP is positioned in an anti-sense orientation to and overlaps with ERCC1. This exceptional type of gene overlap is conserved in the mouse, suggesting that this chromosomal structure has an important biological function [23].

After comprehensively considering these points, we conducted a case-control study to further explore the potential relationship between the polymorphisms of the genes located on 19q13.2–3 or those that are involved in NER and the risk of CBP in a Chinese occupational population. Finally, we identified some valid biomarkers to predict the risk of CBP, which may help protect the health of the population that is occupationally exposed to benzene.

## Materials and Methods

### Study subjects

The study population has previously been exhaustively described [24]. In brief, we recruited 102 CBP patients from several major factories in Shenyang, China as our cases. Benzene poisoning was diagnosed from 1986 to 2011 by the local authorized Occupational Disease Diagnostic Team, China, and includes (a) total WBC counts <4000/μl or WBC counts between 4000 and 4500/μl and platelet counts <80,000/μl, with repeated confirmation of these counts after a few months in a peripheral blood examination; (b) documented benzene exposure as a result of employment in the factory for at least 6 months; and (c) the exclusion of other known causes of abnormal blood counts, such as chloromycetin use and ionizing radiation. The medical records of these patients were independently reviewed by at least two hemopathologists, particularly those with WBC counts >3500 to confirm the CBP diagnosis. Of the 112 eligible patients, 102 (90%) agreed to participate in this study. The diagnostic criteria for occupational CBP are provided by the Ministry of Health, China. Two hundred four healthy workers from the same factories who had been occupationally exposed to similar amounts of benzene in the work environment as the cases were selected as the controls. The cases and controls were matched for age, sex and exposure duration. Each participant donated 2 ml of venous blood and their demographic data were recorded. The intensity of benzene exposure (milligrams per cubic meter) for the patients was used as the benzene exposure level in the workplace while the patients were being diagnosed; the intensity of benzene exposure for the controls was used as the current level and monitored by organic vapor passive dosimetry badges during the collection of the blood samples. The subjects were administered a rigorous physical examination in the Shenyang Occupational Disease Hospital.

### Ethics Statement

The protocol and consent form were approved by the Institutional Review Board of China Medical University prior to the study. Informed consent was obtained from each of the participants after a detailed explanation of the nature and possible consequences of the study. Each participant donated 2 ml venous blood only after written informed consent was obtained and their demographic data (ethnic background, smoking status, alcohol consumption, protective measures, medical history and occupational history, such as work unit, type of work and exposure duration) were recorded in detailed questionnaires. All activities involving human subjects were performed under full compliance with the government’s policies and the Declaration of Helsinki.

### DNA preparation

Venous blood (2 ml) was drawn from each subject and collected with a folic acid sodium anti-coagulant. The subjects’ DNA was routinely extracted from these samples by phenol chloroform extraction, which has been described elsewhere.

### SNaPshot analysis

In our experiments, XPB (rs4150441, G>A), XPC (rs2228001, A>C and rs2279017, C>A), XPF (rs4781560, T>C), XRCC1 (rs25489, G>A) and PPP1R13L (rs1005165, C>T) were detected by multiplex PCR amplification and the probe primer extension SNaPshot reaction method according to the professional recommendation of Invitrogen Company. The procedure is described below.

#### Primer design

Forward and reverse primers used in the multiplex PCR reaction and SNaPshot primers used as probes designed for the study are provided in Table 1 and Table 2. All primers for the reactions were designed using Primer 5 software.

**Table 1:** Primer sequences used for multiplex PCR reaction.

SNPs	Primers	Sequence
**XRCC1 (rs25489)**	Forward primer	5’-CCCCAGTGGTGCTAACCTAA-3’
	Reverse primer	5’-AGGATCTTCCCCAGCTCCT-3’
**PPP1R13L (rs1005165)**	Forward primer	5’-TGCCCCAATTCTGGAGTAGG-3’
	Reverse primer	5’-CGGCACGTGGACACAGATT-3’
**XPB (rs4150441)**	Forward primer	5’-CAAAAGCATGGTGCTGAGTG-3’
	Reverse primer	5’-CCACTTCTGGCAACCACTGA-3’
**XPC (rs2279017)**	Forward primer	5’-TCAGCTGGACACAGCCAATAG-3’
	Reverse primer	5’-GTAAGGGCAGCATCAGAAGG-3’
**XPC (rs2228001)**	Forward primer	5’-GGCCCAAGAAGACCAAAAG-3’
	Reverse primer	5’-CGTGCATGCTGCCTCAGTT-3’
**XPF (rs4781560)**	Forward primer	5’-CCCTCAGTTACGAGCCTCAA-3’
	Reverse primer	5’-CCTTGGCTGTTGAGGGATTT-3’

**Table 2:** Probe primer sequences used for the SNaPshot extension reaction.

SNPs	Size	Direction	Sequence (5’→3’)
XRCC1 (rs25489)	40	Forward	TTTTTTTTTTTTTTTTTTTTTTTAGTGCCAGCTCCAACTC
PPP1R13L (rs1005165)	35	Forward	TTTTTTTTTTTTTTTTTTGAATGCAGTCGGGTCAC
XPB (rs4150441)	65	Forward	TTTTTTTTTTTTTTTTTTTTTTTTTTTTTTTTTTTTTTTTTTTTTTCCTCAAAAGCTTCCATAGT
XPC (rs2279017)	50	Reverse	TTTTTTTTTTTTTTTTTTTTTTTTTTTTTTTTTTCTGACTTGCTCACCCG
XPC (rs2228001)	45	Forward	TTTTTTTTTTTTTTTTTTTTTTTTTTTCACCTGTTCCCATTTGAG
XPF (rs4781560)	55	Forward	TTTTTTTTTTTTTTTTTTTTTTTTTTTTTTTAATATTACAGTTAGATAGAGCCAC

#### PCR amplification

Multiplex PCR amplification was performed from 50 ng of genomic DNA in a final volume of 25 μl containing 2.5 μl of 10×PCR buffer, 0.8 μl of 50 mM MgCl_2_, 0.5 μl of 10 mM dNTP, 0.2 μl of Platinum® Taq DNA-polymerase, and 1 μl of a 5 μM stock of each primer. The PCR cycling conditions were 1 cycle of 94°C for 5 minutes; 33 cycles of 94°C for 30 seconds, 56°C for 30 seconds, and 72°C for 30 seconds; and 1 cycle of 72°C for 5 minutes. The multiplex PCR amplicons were analyzed on 3% (w/v) agarose gels before being treated with an Exo/SAP master mix containing 20 U/μl of Exonuclease-I (Fermentas) and 1 U/μl of Shrimp Alkaline Phosphatase (Fermentas) to remove the unincorporated primers and dNTPs (Invitrogen). The PCR product (2 μl) was incubated with 0.5 μl of the Exo/SAP master mix for 1 hour at 37°C followed by 15 minutes at 75°C to inactivate the enzyme. The second step consisted of a multiplex single-base primer extension reaction according to the manufacturer’s protocol. The final reaction volume was 3 μl containing 1 μl of the treated first-step PCR reaction, 1.5 μl of the SNaPshot ready reaction premix containing fluorescent dideoxy nucleotides (A = dR6G, green; C = dTAMRA, black; G = dR110, blue; T = dROX, red), and 0.5 μl of the probe primers. The reaction was performed under stringent conditions (25 cycles of 96°C for 10 seconds, 51°C for 5 seconds, and 60°C for 30 seconds). An aliquot of the SNaPshot extension reaction (5 μl) was then treated with 0.3 μl 1U/μl of SAP for 1 hour at 37°C followed by 15 minutes at 75°C to inactivate the enzyme before the third step of capillary electrophoresis.

#### Capillary electrophoresis

The fluorescence and size of the extended products were determined by capillary electrophoresis on a 3730 genetic analyzer (Applied Biosystem) using a POP-7 polymer. Before being loaded onto the genetic analyzer, an aliquot of the treated SNaPshot multiplex extension reaction (1 μl) was mixed with 8.8 μl of Hidi-formamide (Applied Biosystems) and 0.2 μl of the size standard (GeneScan-120 LIZ ladder, Applied Biosystem). The data were analyzed using GeneMapper v4.0 and specific parameters.

### TaqMan-MGB analysis

XRCC1 (rs25487, A>G, assay ID is C_622564_10, part number is 4351379; rs1799782, C>T, assay ID is C_11463404_10, part number is 4351379) and CD3EAP (rs967591, G>A, assay ID is C_8713992_1, part number is 4351379) were analyzed by TaqMan^®^ sequencing on an ABI 7500 Real-time PCR system (ABI, US, Stagapore). All PCR reagents were purchased from ABI Company.

#### PCR amplification

The PCR reactions were performed in a 20 μl reaction mixture: 10.0 μl of Premix Ex Taq^TM^, 0.4 μl of each probe and primer, and 2 μl of DNA (l0 ng/μl). The PCR reaction included an initial step at 95°C for 10 min, denaturation at 95°C for 5 s and extension at 60°C for 34 s.

#### Allelic discrimination plate read and analysis

After PCR amplification, an endpoint plate read was performed using an Applied Biosystems Real-Time PCR System. The Sequence Detection System (SDS) Software uses the fluorescence measurements made during the plate read to plot the fluorescence (Rn) values based on the signals from each well. The plotted fluorescence signals indicate the alleles that are present in each sample.

### Statistical analysis

All statistical analyses were performed with SPSS 19.0. To ensure a good fit to the Hardy-Weinberg equilibrium, the linkage disequilibrium (LD) for each SNP was tested using the Haploview Software (version 4.1, Broad Institute). A Fisher’s exact test or Chi-squared (χ^2^) test was selected to compare the frequencies of the different genetic polymorphisms between the cases and controls. The χ^2^ test was also used to evaluate the association between genetic polymorphisms and the risk of CBP. The analyses for the homogeneity of the odds ratios (OR) were performed using the Breslow–Day method. The OR and 95% confidence interval (95% CI) obtained from the multinomial logistic regression were used to analyze the association of the genetic polymorphisms with CBP after adjusting for potential confounders, including cigarette smoking, alcohol consumption, gender and the duration of benzene exposure. The frequency distribution of the haployptes was calculated by χ^2^ analysis. A two-tailed *P*-value < 0.05 was considered statistically significant.

## Results

### Demographics of the cases and controls

A summary of selected characteristics of the subjects is shown in our previous publication [24]. In brief, the 102 CPB patients used as cases were characterized by a median age of 37.5 (range: 18.0–63.0) and an exposure duration of 10.0 (range: 2.0–32.0), and the 204 healthy people used as controls were characterized by a median age of 36.0 (range: 23.0–62.0) and an exposure duration of 10.0 (range: 1.0–35.0). More female participants than male participants (79.41% and 20.59%, respectively) were involved in this survey. A higher proportion of the cases were smokers than the controls (12.75% vs. 5.39%, *P* = 0.024), and the distribution of the other factors between the cases and controls were not significantly different.

### Genetic polymorphisms of XRCC1, PPP1R13L, CD3EAP, XPB, XPC and XPF

The frequencies of the XRCC1, PPP1R13L, CD3EAP, XPB, XPC and XPF genotypes in the cases and controls are presented in Table 3. For XRCC1 rs25487, the allele distribution was significantly different between the cases and controls (*P* <0.01). In contrast, there were no differences in the other SNPs (*P*>0.05).

**Table 3:** Association between single nucleotide polymorphisms of PPP1R13L, XRCC1, XPC, XPF, XPB/ERCC3 and CD3EAP and the risk of CBP.

SNPs	Cases^a^		Controls^a^		OR(95% CI)	*P*	OR_adj_(95% CI)^b^	*P*
	n	%	n	%				
XRCC1 rs25487								**0.000**
GG	11	10.89	99	49.01	1.000		1.000	-
GA	40	39.60	71	35.15	5.070(2.435–10.559)	**0.000**	5.431(2.553–11.552)	**0.000**
AA	50	49.50	32	15.84	14.063(6.545–30.214)	**0.000**	14.898(6.781–32.732)	**0.000**
GA+AA	90	89.11	103	51.00	7.864(3.968–15.587)	**0.000**	8.379(4.135–16.978)	**0.000**
XRCC1 rs25489								0.949
GG	85	84.16	170	.85.00	1.000	-	1.000	-
GA+AA	16	15.84	30	15.00	1.067 (0.551–2.064)	0.848	1.041(0.533–2.034)	0.899
XRCC1 rs1799782								0.078
CC	51	50.00	119	59.20	1.000		1.000	-
CT	34	33.33	65	32.34	1.221(0.719–2.071)	0.460	1.224(0.717–2.088)	0.459
TT	17	16.67	17	8.46	2.333(1.104–4.930)	**0.024**	2.002(0.922–4.348)	0.079
CT+TT	51	50.00	82	40.80	1.451(0.889–2.344)	0.127	1.411(0.868–2.294)	0.164
PPP1R13L rs1005165								0.765
CC	34	33.66	66	33.00	1.000	-	1.000	-
CT	40	39.60	87	43.50	0.892(0.511–1.559)	0.689	0.938(0.532–1.654)	0.825
TT	27	26.73	47	23.50	1.115(0.595–2.091)	0.734	1.157(0.607–2.204)	0.657
CT+TT	67	66.34	134	67.00	0.971(0.585–1.612)	0.908	1.019(0.608–1.707)	0.944
CD3EAP rs967591								0.701
GG	32	32.32	70	34.65	1.000		1.000	-
GA	38	38.38	82	40.60	1.014(0.574–1.789)	0.963	0.937(0.526–1.669)	0.544
AA	29	29.29	50	24.75	1.269(0.683–2.358)	0.451	0.780(0.414–1.468)	0.445
GA+AA	67	67.68	132	65.35	1.110(0.666–1.851)	0.688	0.870(0.518–1.463)	0.449
XPB/ERCC3 rs4150441								0.128
GG	27	26.73	77	38.50	1.000		1.000	-
GA	57	56.44	94	47.00	1.729(1.000–2.992)	**0.049**	1.672(0.959–2.912)	0.070
AA	17	16.83	29	14.50	1.672(0.796–3.511)	0.173	1.638(0.772–3.474)	0.198
GA+AA	74	73.27	123	61.50	1.716(1.015–2.900)	**0.043**	1.668(0.981–2.836)	0.058
XPC rs2279017								0.287
GG	44	43.56	72	36.00	1.000	-	1.000	-
GT	46	45.54	95	47.50	0.792(0.474–1.325)	0.375	0.731(0.432–1.237)	0.243
TT	11	10.89	33	16.50	0.545(0.250–1.188)	0.124	0.487(0.219–1.087)	0.079
GT+TT	57	56.44	128	64.00	0.729 (0.447–1.187)	0.203	0.672(0.407–1.108)	0.117
XPC rs2228001								0.579
AA	45	44.55	78	39.00	1.000		1.000	-
AC	45	44.55	94	47.00	0.830(0.498–1.383)	0.474	1.264(0.562–2.844)	0.571
CC	11	10.89	28	14.00	0.681(0.310–1.497)	0.338	1.639(0.721–3.728)	0.238
AC+CC	56	55.45	122	61.00	0.796(0.490–1.292)	0.355	1.439(0.665–3.114)	0.356
XPF rs4781560								0.614
TT	60	59.41	123	61.50	1.000		1.000	-
TC	35	34.65	70	35.00	1.025(0.616–1.707)	0.924	0.523(0.162–1.694)	0.280
CC	6	5.94	7	3.50	1.757(0.566–5.457)	0.324	0.500(0.159–1.576)	0.237
TC+CC	41	40.59	77	38.50	1.092(0.670–1.779)	0.725	0.508(0.104–1.573)	0.240

a: The data are missing because the sequence could not be amplified.

b: The ORs were adjusted for potential confounding variables, including age, sex, exposure duration, smoking and alcohol consumption.

### The effect of genetic polymorphisms on the risk of CBP were modified by the subjects’ lifestyles

A higher proportion of the cases carry the XRCC1 rs25487 AA genotype than the controls (49.50% vs. 15.84%, respectively) and the risk of CBP was correspondingly increased (OR = 14.063; 95% CI: 6.545–30.214; *P* < 0.001) compared to individuals carrying the GG genotype. The XRCC1 rs25487 AA genotype also seemed to be related to a higher risk of CBP after stratifying by smoking, alcohol consumption, gender and exposure duration (OR_adj_ = 14.898; 95% CI: 6.781–32.732; *P* < 0.001) (Tables 3–7). The proportion of the XRCC1 rs1799782 TT genotype was higher in the cases than in the controls (16.67% vs. 8.46%, respectively) (Table 3). Subjects carrying the rs1799782 TT genotype had a higher risk of CBP (OR = 2.333; 95% CI: 1.014–4.930; *P* = 0.024) and the relationship was confined to alcohol drinkers (OR = 8.000; 95% CI: 1.316–48.645; *P* = 0.022), males (OR = 9.333; 95% CI: 1.593–54.672; *P* = 0.019), and an exposure duration of less than 12 years (OR = 2.612; 95% CI: 1.048–6.510; *P* = 0.035). Moreover, a clear relationship still existed for exposure duration after adjustment (Tables 5, 6 and 7). There was a high risk of CBP for individuals carrying the XPB rs4150441 GA genotype (OR = 1.729; 95% CI: 1.000–2.992; *P* = 0.049) and the GA+AA genotypes (OR = 1.716; 95% CI: 1.015–2.900; *P* = 0.043) compared to those carrying the GG genotype. However, the PPP1R13L rs1005165 T allele (CT genotype, TT genotype and CT+TT genotypes) and CD3EAP rs967591 GA and GA+AA genotypes exhibited a protective role against CBP development in males (*P*<0.05) (Table 6). There was no association between the risk of CBP and XPC and XPF polymorphisms in the present study (*P* > 0.05).

**Table 4:** The relationship between the single nucleotide polymorphisms and the risk of CBP, stratified by smoking status.

Groups	Smoking		OR(95% CI)	No Smoking		OR(95% CI)	OR_adj_(95% CI)
	Cases(%)^a^	Controls(%)^a^		Cases(%)^a^	Controls(%)^a^		
XRCC1 rs25487							
GG	2(15.38)	4(36.36)	1.00	9(10.23)	75(49.74)	1.000	1.000
GA	2(15.38)	5(45.45)	4.800(0.540–42.632)	38(43.18)	66(33.55)	6.077(2.754–13.412)**	4.958(2.382–10.319) **
AA	9(69.23)	2(18.18)	54.000(6.584–442.902)**	41(46.59)	30(15.71)	14.426(6.290–33.085)**	13.708(6.291–29.870) **
GA+AA	11(84.61)	7(63.63)	18.857(3.357–105.933) **	79(89.77)	96(50.26)	8.686(4.121–18.308)**	7.808(3.916–15.566) **
XRCC1 rs25489							
GG	12(92.31)	8(72.73)	1.000	73(82.95)	162(85.71)	1.000	1.000
GA+AA	1(7.69)	3(27.27)	0.222(0.019–2.533)	15(17.05)	27(14.29)	1.233(0.619–2.456)	1.057(0.548–2.038)
XRCC1 rs1799782							
CC	4(30.77)	8(72.73)	1.000	47(52.81)	111(58.42)	1.000	1.000
CT	3(23.08)	2(18.18)	3.000(0.348–25.870)	31(34.83)	63(33.16)	1.162(0.671–2.012)	1.233(0.726–2.094)
TT	6(46.15)	1(9.09)	12.00(1.053–136.794)	11(12.36)	16(8.42)	1.624(0.701–3.761)	2.135(0.998–4.564)
CT+TT	9(69.23)	3(27.27)	6.000(1.018–35.374)	42(47.19)	79(41.58)	1.256(0.757–2.083)	1.427(0.882–2.309)
PPP1R13L rs1005165							
CC	6(46.15)	4(36.36)	1.000	28(31.82)	62(32.80)	1.000	1.000
CT	2(15.38)	4(36.36)	0.333(0.040–2.769)	38(43.18)	83(43.92)	1.014(0.563–1.826)	0.932(0.531–1.637)
TT	5(38.46)	3(27.27)	1.111(0.164–7.506)	22(25.00)	44(23.28)	1.107(0.561–2.183)	1.108 (0.584–2.100)
CT+TT	7(53.85)	7(63.64)	0.667(0.129–3.446)	60(68.18)	127(67.20)	1.046(0.609–1.798)	1.001(0.559–1.671)
CD3EAP rs967591							
GG	5(38.46)	4(36.36)	1.000	27(31.40)	66(34.55)	1.000	1.000
GA	2(15.38)	4(36.36)	0.400(0.047–3.424)	36(41.86)	78(40.84)	1.128(0.621–2.050)	1.044(0.589–1.849)
AA	6(46.15)	3(27.27)	1.600(0.237–10.809)	23(26.74)	47(24.61)	1.196(0.612–2.338)	1.235(0.657–2.324)
GA+AA	8(61.54)	7(63.64)	0.914(0.174–4.811)	59(68.60)	125(65.45)	1.154(0.669–1.989)	1.128(0.673–1.891)
XPB/ERCC3 rs4150441							
GG	1(7.69)	5(45.45)	1.00	26(29.54)	72(38.10)	1.000	1.000
GA	9(69.23)	6(54.55)	7.500(0.692–81.248)	48(54.55)	88(46.56)	1.510(0.854–2.671)	1.681(0.971–2.908)
AA	3(23.08)	0(0)	-	14(15.91)	29(15.34)	1.337(0.613–2.916)	-
GA+AA	12(92.31)	6(54.55)	10.00(0.944–105.921)	62(70.46)	117(61.9)	1.467(0.852–2.528)	1.657(0.982–2.797)
XPC rs2279017							
GG	3(23.08)	1(9.09)	1.000	41(46.59)	71(37.57)	1.000	1.000
GT	8(61.54)	7(63.64)	0.381(0.032–4.550)	38(43.18)	88(46.56)	0.748(0.435–1.284)	0.723(0.427–1.224)
TT	2(15.38)	3(27.27)	0.222(0.012–3.979)	9(10.23)	30(15.87)	0.520(0.225–1.201)	0.487(0.218–1.089)
GT+TT	10(76.92)	10(90.91)	0.333(0.029–3.775)	47(53.41)	118(62.43)	0.690(0.413–1.151)	0.666(0.404–1.097)
XPC rs2228001							
AA	3(23.08)	1(9.09)	1.000	42(47.73)	77(40.74)	1.000	1.000
AC	8(61.54)	7(63.64)	0.381(0.032–4.550)	37(42.05)	87(46.03)	0.780(0.455–1.335)	0.752(0.445–1.271)
CC	2(15.38)	3(27.27)	0.222(0.012–3.979)	9(10.23)	25(13.23)	0.660(0.282–1.544)	0.604(0.268–1.363)
AC+CC	10(76.92)	10(90.91)	0.333(0.029–3.775)	46(52.27)	112(59.26)	0.753(0.453–1.253)	0.724(0.441–1.190)
XPF rs4781560							
TT	11(84.62)	7(63.64)	1.000	49(55.68)	116(61.38)	1.000	1.000
TC	2(15.38)	4(36.36)	0.318(0.046–2.223)	33(37.50)	66(34.92)	1.184(0.693–2.021)	1.071(0.641–1.788)
CC	0	0	-	6(6.82)	7(3.70)	2.029(0.649–6.347)	-
TC+CC	2(15.38)	4(36.36)	0.318(0.046–2.223)	39(44.32)	73(38.62)	1.265(0.758–2.111)	1.147(0.701–1.877)

a: The data are missing because the sequence could not be amplified.

**P < 0.01.

**Table 5:** The relationship between the single nucleotide polymorphisms and the risk of CBP, stratified by alcohol consumption.

**Groups**	Alcohol consumption		OR(95% CI)	No Alcohol consumption		OR(95% CI)	OR_adj_(95% CI)
	Cases(%)^a^	Controls(%)^a^		Cases(%)^a^	Controls(%)^a^		
XRCC1 rs25487							
GG	3(18.75)	12(50.00)	1.000	8(9.41)	87(48.88)	1.000	1.000
GA	2(12.50)	8(33.33)	1.000(0.135–7.392)	38(44.71)	63(35.39)	6.560(2.865–15.020)**	5.048(2.417–10.544) **
AA	11(68.75)	4(16.67)	11.000(1.998–60.572)**	39(45.88)	28(15.73)	15.147(6.335–36.220)**	14.217(6.547–30.870) **
GA+AA	13(81.25)	12(50.00)	4.333(0.978–19.202)	77(90.59)	91(51.12)	9.202(4.197–20.177)**	8.007(4.018–1 958) **
XRCC1 rs25489							
GG	14(87.50)	18(75.00)	1.000	71(83.53)	152(86.36)	1.000	1.000
GA+AA	2(12.50)	6(25.00)	0.429(0.075–2.457)	14(16.47)	24(13.64)	1.249(0.610–2.557)	1.049(0.544–2.025)
XRCC1 rs1799782							
CC	7(43.75)	16(66.67)	1.000	44(51.16)	103(58.19)	1.000	1.000
CT	2(12.50)	6(25.00)	0.762(0.122–4.751)	32(37.21)	59(33.33)	1.270(0.728–2.215)	1.214(0.714–2.064)
TT	7(43.75)	2(8.33)	8.000(1.316–48.645)*	10(11.63)	15(8.47)	1.561(0.651–3.742)	2.220(1.045–4.713)*
CT+TT	9(56.25)	8(33.33)	2.571(0.699–9.457)	42(48.84)	74(41.81)	1.329(0.792–2.230)	1.455(0.901–2.350)
PPP1R13L rs1005165							
CC	5(31.25)	8(33.33)	1.000	29(34.12)	58(32.95)	1.000	1.000
CT	7(43.75)	9(37.50)	1.244(0.280–5.529)	33(38.82)	78(44.32)	0.846(0.463–1.547)	0.894(0.511–1.563)
TT	4(25)	7(29.17)	0.914(0.174–4.811)	23(27.06)	40(22.73)	1.150(0.583–2.269)	1.113(0.593–2.086)
CT+TT	11(68.75)	16(66.67)	1.100(0.284–4.267)	56(65.88)	118(67.05)	0.949(0.549–1.641)	0.969(0.583–1.610)
CD3EAP rs967591							
GG	5(31.25)	7(29.17)	1.000	27(32.53)	63(35.39)	1.000	1.000
GA	7(43.75)	9(37.50)	1.089(0.240–4.950)	31(37.35)	73(41.01)	0.991(0535–1.835)	1.004(0.567–1.777)
AA	4(25.00)	8(33.33)	0.700(0.133–3.684)	25(30.12)	42(23.60)	1.389(0.711–2.713)	1.260(0.678–2.340)
GA+AA	11(68.75)	17(70.83)	0.906(0.229–3.585)	56(67.47)	115(64.61)	1.136(0.654–1.974)	1.101(0.660–1.838)
XPB/ERCC3 rs4150441							
GG	3(18.75)	9(37.50)	1.000	24(28.24)	68(38.64)	1.000	1.000
GA	11(68.75)	14(58.33)	2.357(0.512–10.850)	46(54.12)	80(45.45)	1.629(0.903–2.939)	1.712(0.988–2.966)
AA	2(12.50)	1(4.17)	6.000(0.390–92.277)	15(17.65)	28(15.91)	1.518(0.695–3.314)	1.691(0.804–3.556)
GA+AA	13(81.25)	15(62.50)	2.600(0.578–11.687)	61(71.76)	108(61.36)	1.600(0.913–2.805)	1.702(1.007–2.877)
XPC rs2279017							
GG	7(43.75)	6(25.00)	1.000	37(43.53)	66(37.50)	1.000	1.000
GT	8(50.00)	15(62.50)	0.457(0.114–1.831)	38(44.71)	80(45.45)	0.847(0.485–1.480)	0.778(0.464–1.303)
TT	1(6.25)	3(12.50)	0.286(0.023–3.523)	10(11.76)	30(17.05)	0.595(0.262–1.352)	0.552(0.253–1.203)
GT+TT	9(56.25)	18(75.00)	0.429(0.111–1.657)	48(56.47)	110(62.50)	0.778(0.447–1.187)	0.720(0.441–1.173)
XPC rs2228001							
AA	7(43.75)	7(29.17)	1.000	38(44.71)	71(40.34)	1.000	1.000
AC	8(50.00)	15(62.50)	0.533(0.138–2.066)	37(43.53)	79(44.89)	0.875(0.503–1.524)	0.815(0.488–1.361)
CC	1(6.25)	2 (8.33)	0.500(0.036–6.862)	10(11.76)	26(14.77)	0.719(0.314–1.646)	0.695(0.315–1.532)
AC+CC	9(56.25)	17(70.83)	0.529(0.141–1.988)	47(55.29)	105(59.66)	0.836(0.496–1.411)	0.786(0.484–1.278)
XPF rs4781560							
TT	10(62.50)	17(70.83)	1.000	50(58.82)	106(60.23)	1.000	1.000
TC	6(37.50)	7(29.17)	1.457(0.381–5.572)	29(34.12)	63(35.80)	0.976(0.561–1.698)	1.034(0.620–1.724)
CC	0	0	-	6(7.06)	7(3.98)	1.817(0.581–5.688)	-
TC+CC	6(37.50)	7(29.17)	1.457(0.381–5.572)	35(41.18)	70(39.77)	1.060(0.626–1.795)	1.106(0.677–1.805)

a: The data are missing because the sequence could not be amplified.

*P < 0.05

**P< 0.01.

**Table 6:** The relationship between the single nucleotide polymorphisms and the risk of CBP, stratified by gender.

**Groups**	Male		OR(95% CI)	Female		OR(95% CI)	OR_adj_(95% CI)
	Cases(%)^a^	Controls(%)^a^		Cases(%)^a^	Controls(%)^a^		
XRCC1 rs25487							
GG	2(9.52)	19(46.34)	1.000	9(11.25)	80(49.69)	1.000	1.000
GA	7(33.33)	13(31.71)	5.115(0.914–28.640)	33(41.25)	58(36.02)	5.057(2.248–11.378)**	5.068(2.433–10.555) **
AA	12(57.14)	9(21.95)	12.667(2.328–68.926)**	38(47.50)	23(14.29)	14.686(6.202–34.773)**	14.207(6.581–30.671)**
GA+AA	19(90.48)	22(53.66)	8.205(1.688–39.874)**	71(88.75)	81(50.31)	7.791(3.647–16.647)**	7.870(3.970–15.603) **
XRCC1 rs25489							
GG	19(95.00)	33(80.49)	1.000	66(81.48)	137(86.16)	1.000	1.000
GA+AA	1(5.00)	8(19.51)	0.217(0.025–1.872)	15(18.52)	22(13.84)	1.415(0.690–2.905)	1.066(0.552–2.057)
XRCC1 rs1799782							
CC	9(42.86)	28(68.29)	1.000	42(51.85)	91(56.88)	1.000	1.000
CT	6(28.57)	11(26.83)	1.697(0.488–5.902)	28(34.57)	54(33.75)	1.123(0.626–2.016)	1.208(0.712–2.051)
TT	6(28.57)	2(4.88)	9.333(1.593–54.672)*	11(13.58)	15(9.37)	1.589(0.673–3.753)	2.299(1.096–4.821)*
CT+TT	12(57.14)	13(31.71)	2.872(0.969–8.508)	39(48.15)	69(43.12)	1.225(0.716–2.094)	1.448(0.898–2.335)
PPP1R13L rs1005165							
CC	11(55.00)	8(19.51)	1.000	23(28.40)	58(36.48)	1.000	1.000
CT	4(20.00)	18(43.90)	0.162(0.039–0.666)*	36(44.44)	69(43.40)	1.316(0.701–2.468)	0.899(0.518–1.557)
TT	5(25.00)	15(36.59)	0.242(0.062–0.946)*	22(27.16)	32(20.13)	1.734(0.838–3.585)	1.082(0.584–2.006)
CT+TT	9(45.00)	33(80.49)	0.198(0.061–0.640)**	58(71.60)	101(63.52)	1.448(0.810–2.589)	0.972(0.590–1.601)
CD3EAP rs967591							
GG	11(52.38)	8(19.51)	1.000	21(26.92)	62(38.51)	1.000	1.000
GA	4(19.05)	18(43.90)	0.162(0.039–0.666)*	34(43.59)	64(39.75)	1.568(0.822–2.994)	1.014(0.580–1.772)
AA	6(28.57)	15(36.59)	0.291(0.078–1.082)	23(29.49)	35(21.74)	1.940(0.942–3.995)	1.212(0.658–2.232)
GA+AA	10(47.62)	33(80.49)	0.220(0.070–0.698) **	57(73.08)	99(61.49)	1.700(0.940–3.074)	1.204(0.722–2.006)
XPB/ERCC3 rs4150441							
GG	4(20.00)	17(41.46)	1.000	23(28.40)	60(37.74)	1.000	1.000
GA	13(65.00)	20(48.78)	2.763(0.758–10.074)	44(54.32)	74(46.54)	1.551(0.844–2.850)	1.731(1.001–2.996)
AA	3(15.00)	4(9.76)	3.188(0.501–20.298)	14(17.28)	25(15.72)	1.461(0.649–3.290)	1.648(0.785–3.456)
GA+AA	16(80.00)	24(58.54)	2.833(0.804–9.984)	58(71.60)	99(62.26)	1.528(0.856–2.729)	1.714(1.015–2.895)
XPC rs2279017							
GG	9(45.00)	10(24.39)	1.000	35(43.21)	62(38.99)	1.000	1.000
GT	9(45.00)	22(53.66)	0.455(0.138–1492)	37(45.68)	73(45.92)	0.898(0.506–1.592)	0.790(0.473–1.321)
TT	2(10.00)	9(21.95)	0.247(0.042–1.460)	9(11.11)	24(15.09)	0.664(0.278–1.587)	0.541(0.249–1.176)
GT+TT	11(55.00)	31(75.61)	0.582(0.183–1.855)	46(56.79)	97(61.01)	0.840(0.488–1.446)	0.786(0.481–1.285)
XPC rs2228001							
AA	9(45.00)	10(24.39)	1.000	36(44.44)	68(42.77)	1.000	
AC	9(45.00)	22(53.66)	0.455(0.138–1.492)	36(44.44)	72(45.28)	0.944(0.535–1.668)	0.825(0.4951.374)
CC	2(10.00)	9(21.95)	0.247(0.042–1.460)	9(11.11)	19(11.95)	0.895(0.367–2.179)	0.673(0.307–1.474)
AC+CC	11(55.00)	31(75.61)	0.394(0.127–1.224)	45(55.56)	91(57.23)	0.934(0.545–1.602)	0.799(0.492–1.294)
XPF rs4781560							
TT	14(70.00)	29(70.73)	1.000	46(56.79)	94(59.12)	1.000	1.000
TC	5(25.00)	12(29.27)	0.863(0.254–2.932)	30(37.04)	58(36.48)	1.057(0.601–1.859)	1.020(0.611–1.701)
CC	1(5.00)	0(0.00)	-	5(6.17)	7(4.40)	1.460(0.439–4.849)	-
TC+CC	6(30.00)	12(29.27)	1.036(0.322–3.335)	35(43.21)	65(40.88)	1.100(0.640–1.891)	1.089(0.666–1.779)

a: The data are missing because the sequence could not be amplified.

*P< 0.05

**P< 0.01.

**Table 7:** The relationship between the single nucleotide polymorphisms and the risk of CBP, stratified by exposure duration.

Groups	Exposure duration(y)≤12		OR(95% CI)	Exposure duration(y)>12		OR(95% CI)	OR_adj_(95% CI)
	Cases(%)^a^	Controls(%)^a^		Cases(%)^a^	Controls(%)^a^		
XRCC1 rs25487							
GG	6(9.84)	67(51.54)	1.000	5(12.50)	32(44.44)	1.000	
GA	21(34.43)	45(34.62)	5.211(1.950–13.924)**	19(47.50)	26(36.11)	4.677(1.537–14.231)**	4.971(2.380–10.383)**
AA	34(55.74)	18(13.85)	21.093(7.668–58.023)**	16(40.00)	14(19.44)	7.314(2.238–23.909)**	13.551(6.334–28.990)**
GA+AA	55(90.16)	63(48.46)	9.749(3.923–24.223)**	35(87.50)	40(55.56)	5.600(1.967–15.939)**	7.781(3.928–15.411)**
XRCC1 rs25489							
GG	54(88.54)	108(84.38)	1.000	31(77.50)	62(86.11)	1.000	
GA+AA	7(11.46)	20(15.62)	0.700(0.279–1.758)	9(22.50)	10(13.89)	1.800(0.663–4.885)	1.059(0.547–2.051)
XRCC1 rs1799782							
CC	33(53.23)	79(61.24)	1.000	18(45.00)	40(55.56)	1.000	
CT	17(27.42)	39(30.23)	1.044(0.518–2.101)	17(42.50)	26(36.11)	1.453(0.636–3.321)	1.198(0.704–2.039)
TT	12(19.35)	11(8.53)	2.612(1.048–6.510)*	5(12.50)	6(8.33)	1.852(0.499–6.868)	2.332(1.104–4.926)*
CT+TT	29(46.77)	50(38.76)	1.388(0.753–2.560)	22(55.00)	32(44.44)	1.528(0.702–3.324)	1.440(0.891–2.329)
PPP1R13L rs1005165							
CC	20(32.79)	41(32.03)	1.000	14(35.00)	25(34.72)	1.000	
CT	21(34.43)	59(46.09)	0.730(0.351–1.515)	19(47.50)	28(38.89)	1.212(0.505–2.910)	0.900(0.514–1.574)
TT	20(32.79)	28(21.88)	1.464(0.668–3.208)	7(17.50)	19(26.39)	0.658(0.222–1.949)	1.107(0.590–2.074)
CT+TT	41(67.21)	87(67.97)	0.966(0.504–1.852)	26(65.00)	47(65.28)	0.988(0.439–2.223)	0.975(0.587–1.619)
CD3EAP rs967591							
GG	19(31.67)	44(33.85)	1.000	13(33.33)	26(36.11)	1.000	
GA	20(33.33)	56(43.07)	0.827(0.394–1.736)	18(46.15)	26(36.11)	1.385(0.565–3.395)	1.021(0.578–1.804)
AA	21(35.00)	30(23.08)	1.621(0.747–3.518)	8(20.51)	20(27.78)	0.800(0.278–2.300)	1.262(0.680–2.344)
GA+AA	41(68.33)	86(66.15)	1.104(0.574–2.124)	26(66.67)	46(63.89)	1.130(0.497–2.570)	1.114(0.668–1.859)
XPB/ERCC3 rs4150441							
GG	15(24.59)	46(35.94)	1.000	12(30.00)	31(43.06)	1.000	
GA	33(54.10)	61(47.66)	1.659(0.807–3.410)	24(60.00)	33(45.83)	1.879(0.804–4.391)	1.747(1.009–3.026)
AA	13(21.31)	21(16.41)	1.898(0.768–4.690)	4(10.00)	8(11.11)	1.292(0.327–5.097)	1.689(0.796–3.582)
GA+AA	46(75.41)	82(64.06)	1.720(0.867–3.415)	28(70.00)	41(56.94)	1.764(0.776–4.012)	1.738(1.027–2.942)
XPC rs2279017							
GG	26(42.62)	45(35.16)	1.000	18(45.00)	27(37.50)	1.000	
GT	27(44.26)	63(49.22)	0.742(0.383–1.436)	19(47.50)	32(44.44)	0.891(0.391–2.029)	0.797(0.476–1.334)
TT	8(13.11)	20(15.63)	0.692(0.267–1.793)	3(7.50)	13(18.06)	0.346(0.086–1.390)	0.546(0.251–1.189)
GT+TT	35(57.38)	83(64.84)	0.730(0.391–1.362)	22(55.00)	45(62.50)	0.733(0.335–1.607)	0.731(0.449–1.192)
XPC rs2228001							
AA	26(42.62)	47(36.72)	1.000	19(47.50)	31(43.06)	1.000	
AC	27(44.26)	62(48.44)	0.787(0.407–1.521)	18(45.00)	32(44.44)	0.918(0.407–2.067)	0.837(0.502–1.395)
CC	8(13.11)	19(14.84)	0.761(0.293–1.978)	3(7.50)	9(12.50)	0.544(0.131–2.264)	0.683(0.310–1.507)
AC+CC	35(57.38)	81(63.28)	0.781(0.419–1.455)	21(52.5)	41(56.94)	0.836(0.384–1.816)	0.802(0.494–1.303)
XPF rs4781560							
TT	38(62.30)	74(57.81)	1.000	22(55.00)	49(68.06)	1.000	
TC	20(32.79)	48(37.50)	0.811(0.423–1.557)	15(37.50)	22(30.56)	1.519(0.664–3.472)	1.028(0.618–1.710)
CC	3(4.92)	6(4.69)	0.974(0.231–4.110)	3(7.50)	1(1.39)	6.682(0.658–67.883)	1.743(0.564–5.382)
TC+CC	23(37.70)	54(42.19)	0.829(0.444–1.550)	18(45.00)	23(31.94)	1.743(0.786–3.863)	1.098(0.674–1.788)

a: The data are missing because the sequence could not be amplified.

*P < 0.05

**P< 0.01.

### Effects of the Haplotypes on Risk of CBP

Using the Haploview 4.1 program and criteria based on the 95% confidence interval bounds on the D’ values, we performed a combined analysis of 10 SNPs encompassing XRCC1, XPC, XPF, PPP1R13L, CD3EAP and ERCC1; the genotyping data for ERCC1 that was used in the haplotype analysis were from our previous publication [24]. Three haplotype blocks with strong LD in the studied sub-region were identified and the results were presented in Table 8. The association between the haplotypes and CBP risk was assessed for each haplotype block and evaluated by χ^2^ analysis. There were statistically significant differences for the distribution of the XRCC1 rs25487^A^, rs25489^G^ and rs1799782^T^ haplotypes. Compared to those carrying the XRCC1 rs25487^G^, rs25489^G^ and rs1799782^C^ haplotypes, there was an increased risk of CBP with the AGT haplotypes (OR = 15.469, 95% CI: 5.536–43.225, *P*<0.001). In addition, the PPP1R13L, CD3EAP and ERCC1 polymorphisms are in linkage disequilibrium with each other in cancers, and XPC rs2228001 and rs2279017 and XPF rs4781560, which are located in the same region, had significant linkage disequilibrium. However, in the current study, there were no significant associations between these haplotypes and the risk of CBP.

**Table 8:** Association between the haplotypes and the risk of CBP.

Haplotype	Frequency^a^	Groups		OR (95% CI)	*P* ^b^
		Cases (n)	Controls (n)		
XRCC1 rs25487 G>A; XRCC1 rs25489 G>A; XRCC1 rs17998872C>T					
GGC	0.350	32	75	1.000	
AGC	0.295	28	62	1.058(0.576–1.945)	0.855
GGT	0.142	0	44	-	-
AGT	0.129	33	5	15.469(5.536–43.225)	**0.000**
GAC	0.055	0	16	-	**-**
PPP1R13L rs1005165 C>T; CD3EAP rs967591 G>A; ERCC1 rs3212986 G>T^c^; ERCC1 rs11615 C>T^c^						
CGGC	0.061	5	14	1.000	
TAGC	0.399	41	81	1.417(0.477–4.207)	0.528
CGTC	0.276	28	57	1.375(0.450–4.202)	0.575
CGGT	0.178	17	38	1.253(0.389–4.037)	0.706
TAGT	0.030	5	4	3.500(0.662–18.495)	0.210
XPF rs4781560 T>C; XPC rs2228001 A>C; XPC rs2279017 C>A						
TAC	0.484	51	97	1.000	
TCA	0.284	26	60	0.824(0.465–1.460)	0.507
CAC	0.135	16	25	1.217(0.597–2.484)	0.589
CCA	0.076	8	16	0.951(0.381–2.372)	0.914

a: A Frequency of less than 0.03 is not included in the table.

b: The *P* value was obtained using the χ^2^test.

c: The genotyping data that were included in the haplotype analysis were from our previously published study.

## Discussion

It is well known that a valid DNA repair process after exposure to benzene and its metabolites may contribute to reduce the risk of chronic benzene poisoning (CBP). Mounting scientific evidence has shown that occupational exposure to benzene induces genetic damage, mainly due to chromosomal aberrations, disordered DNA structure and oxidative damage [25–27]. Although some epidemiological studies on the association between the risk of CBP and polymorphism in DNA repair genes have been performed, the conclusions are controversial.

XRCC1, a central scaffolding protein involved in BER, is thought to be a critical element of an individual’s susceptibility to benzene genotoxicity [28]. Three coding polymorphisms, which are precisely located on these interacting domains, are identified in the XRCC1 gene, including rs25487 G>A (Arg→Gln), rs25489 G>A (Arg→His) and rs1799782 C>T (Arg→Trp) [29]. We hypothesized that these variations may affect the normal function of XRCC1 to repair benzene-induced DNA damage. We found that two XRCC1 SNPs (rs25487 and rs1799782) showed a close correlation with a higher risk of CBP. Recently, the significance of these two polymorphisms has been extensively emphasized in several cancers. The XRCC1 rs1799782 TT genotype was found to be associated with an increased risk of lung [30], esophageal [31], and cervical cancer [32] in a Chinese population, and the rs25487 AA genotype was linked to an increased risk of breast cancer among women [33] and pancreatic cancer [34] in a Chinese population. These two SNPs are also reported to be linked to the repair of benzene-induced DNA damage. A study of Thai laboratory workers who were occupationally exposed to benzene indicated that the participants carrying the XRCC1 rs25487 AA or GA genotypes had a reduced DNA repair capacity compared to those with the GG genotype [35]. However, our results were not in accord with a study performed in south China, which suggested that individuals carrying the XRCC1 rs1799782 TT genotype exhibited a reduced risk of CBP comparing to other genotypes. The XRCC1 rs25487 AA genotype was not relevant to the risk of CBP in the occupational population [11]. The possible explanation for the different conclusions is that the subjects involved in these two studies may have different genetic backgrounds due to their different geographical location in China. In addition, the sample size and constituent ratio may also explain the distinction. Although the results are not consistent, XRCC1 still has a critical function in repairing the damage caused by CBP and the XRCC1 polymorphisms may become a valid biomarker to predict the susceptibility of CBP. Furthermore, our study indicated that XRCC1 rs25487 significantly predicted the benzene toxicity, and an increased risk still existed after stratification. These meaningful results suggest that XRCC1 rs25487 G>A may be used as a valid biomarker to help identify individuals who are at high risk for CBP and prevent occupational diseases.

In our previous study, we found that ERCC1, an endonuclease in the NER system, which is located on 19q13.3, could reflect an association with the capacity to repair the benzene-induced DNA damage, and ERCC1 polymorphisms at codon 118 were associated with a difference in CPB risk. We hypothesize that other genes that are located on 19q13.2–3 could also have an effect on the risk of CBP. Therefore, we also determined whether PPP1R13L and CD3EAP polymorphisms were valid biomarkers, in addition to XRCC1. Several epidemiological studies had demonstrated that PPP1R13L rs1005165 and CD3EAP rs967591 were related to an individual’s susceptibility to cancer [23, 36, 37]. One study detected polymorphisms in several genes related to apoptosis in patients with early stage non-small-cell lung cancer and found that PPP1R13L rs1005165 was associated with a difference in the survival rate [36]. The evidence indicated that rs967591G>A affects CD3EAP expression and thus impacted the survival of patients with early stage non-small-cell lung cancer [23]. Over-expression of PPP1R13L suppressed the function of p53, which is a well-known tumor suppressor protein that plays a central role in mediating cellular responses to DNA damage [38, 39]. It is still an interesting question of whether PPP1R13L could affect the susceptibility to CBP, and additional studies are needed to further explore the role of PPP1R13L in cellular apoptosis and DNA repair. Very little has been confirmed about the function of CD3EAP. However, the structure of the CD3EAP coding region partially overlaps with the 3’-untranslated region of ERCC1, suggesting that CD3EAP may exert some of its functions by affecting ERCC1. Interestingly, CD3EAP has a separate reverse complementary overlap with PPP1R13L, and both of them share a promoter region. Thus, we speculate PPP1R13L and CD3EAP, together with ERCC1, may have a key role in the ability of the DNA repair machinery to repair the damage caused by benzene and its metabolites. Furthermore, a relationship between the PPP1R13L and CD3EAP polymorphisms with the risk of CBP has been observed in males, which further verifies the likelihood of the above explanation.

NER is thought to repair most DNA damage, including the DNA adducts caused by benzene and its metabolites BQ and HQ [40]. XPB, XPC and XPF are involved in the NER pathway and play an important role in repairing bulky DNA adducts. We found that the XPB rs4150441 GA and GA +AA genotypes may be associated to an increased risk of CBP. A related study suggested that XPB rs4150441 could predict an individual’s susceptibility to benzene-induced hematotoxicity at relatively low levels of benzene exposure [3]. Although polymorphisms in other NER genes have also been found be associated with benzene poisoning susceptibility in Chinese workers, we did not find any evidence indicating that XPC and XPF polymorphisms could affect an individual’s sensitivity to CBP in our current data.

A haplotype analysis was conducted in our current study to obtain much more information from a number of tightly linked SNPs. XRCC1 (rs25487, rs25489 and rs1799782) PPP1R13L rs1005165, CD3EAP rs967591, and ERCC1 (rs3212986, rs11615) are located in close proximity on chromosome 19q13, and the linkage disequilibrium of these SNPs was considered more important for indicating an individual’s DNA repair capacity. The results showed that the carriers of the XRCC1 rs25487^A^, rs25489^G^ and rs1799782^T^ haplotypes had a higher risk of CBP than those carrying the rs25487^G^, rs25489^G^ and rs1799782^C^ haplotypes. These results better explain the contribution of XRCC1 rs25487 and rs1799782 to CBP. Furthermore, ERCC1, CD3EAP and PPP1R13L, which are all located on 19q13.2–3, were identified as a high risk region that predicts the development of the disease. However, in our current study, the haplotype analysis of these four SNPs did not show a relationship between any of the haplotypes and the risk of CBP in the Chinese population. A haplotype analysis of three SNPs (XPF rs4781560, XPC rs2279017 and rs2228001) was also performed in our study, but no meaningful result was observed. It indicated that many more studies would be necessary to identify the possible mechanisms.

In conclusion, we found that the variant XRCC1 rs25487 and rs1799782 alleles may contribute to predicting the risk of CBP. We focused on the functions of these genes, located in chromosome 19q13.2–3, to identify valid biomarkers to predict the risk of CBP susceptibility. We found, for the first time, that the entire 19q13.2-19q13.3 region, containing XRCC1, PPP1R13L, CD3EAP and ERCC1, may have a particular association with CBP in occupationally exposed Chinese populations through genetic variation. Although there are several limitations in the present study, some of these SNPs showed a weak association with the risk of CBP. An increased sample size, improved experimental design and methods, systematic follow-up and deeper mechanistic study would be of interest. Other SNPs related to the DNA repair capacity that may affect the susceptibility to CBP are the subjects of further research. All valid measures will contribute to protect the individuals who are susceptible to CBP, and provide a possibility for early diagnosis and treatment.

## Supporting Information

S1 Fig Part of SNaPshot QC genotyping results. GeneMapper electropherograms of SNaPshot reactions. Plots of size (nt) versus relative fluorescence units (rfus) for 10 DNA samples exhibiting variations at the SNP sites (PPP1R13L rs1970764 and XPF rs2020955 were not chose for our data analysis at last). The x axis represents the size (bp) of the primer pair with the incorporated nucleotides, while the y axis corresponds to the relative fluorescent units of the peak. Each fluorescent dye corresponds to a different nucleotide: blue represents G, green represents A, red represents T, and black represents C. The orange peak represents the size standard.
(PDF)

S1 Supporting Information Illustration of Capillary Electrophoresis. (PDF)

S1 Table Description of the missing data. (DOCX)

## References

[1] Rodriguez B, Yang Y, Guliaev AB, Chenna A, Hang B. Benzene-derived N2-(4-hydroxyphenyl)-deoxyguanosine adduct: UvrABC incision and its conformation in DNA. Toxicol Lett. 2010; 193:26–32. 10.1016/j.toxlet.2009.12.005 20006688

[2] Capleton AC, Levy LS. An overview of occupational benzene exposures and occupational exposure limits in Europe and North America. Chem Biol Interact. 2005;153: 43–53. 15935799 10.1016/j.cbi.2005.03.007

[3] Hosgood HD 3rd, Zhang L, Shen M, Berndt SI, Vermeulen R, Li G, et al Association between genetic variants in VEGF, ERCC3 and occupational benzene haematotoxicity. Occup Environ Med. 2009;66(12):848–853. 10.1136/oem.2008.044024 19773279 PMC2928224

[4] Lan Q, Zhang L, Li G, Vermeulen R, Weinberg RS, Dosemeci M, et al Hematotoxicity in workers exposed to low levels of benzene. Science. 2004; 306: 1774–1776. 15576619 10.1126/science.1102443 PMC1256034

[5] Seaton MJ, Schlosser PM, Bond JA, Medinsky MA. Benzene metabolism by human liver microsomes in relation to cytochrome P450 2E1 activity. Carcinogenesis. 1994; 15: 1799–1806. 7923572 10.1093/carcin/15.9.1799

[6] Li Y, Kuppusamy P, Zweier JL, Trush MA. ESR evidence for the generation of reactive oxygen species from the copper-mediated oxidation of the benzene metabolite, hydroquinone: role in DNA damage. Chem Biol Interact. 1995; 94: 101–120. 7828218 10.1016/0009-2797(94)03326-4

[7] Kolachana P, Subrahmanyam VV, Meyer KB, Zhang L, Smith MT. Benzeneand its phenolic metabolites produce oxidative DNA damage in HL60 cells in vitro and in the bone marrow in vivo. Cancer Res. 1993; 53:1023–1026. 8439949

[8] Andreoli C, Leopardi P, Crebelli R. Detection of DNA damage in human lymphocytes by alkaline single cell gel electrophoresis after exposure to benzene or benzene metabolites. Mutat Res. 1997; 377:95–104. 9219584 10.1016/s0027-5107(97)00065-1

[9] Amin RP, Witz G. DNA-protein crosslink and DNA strand break formation in HL-60 cells treated with trans,trans-muconaldehyde, hydroquinone and their mixtures. Int J Toxicol. 2001;20:69–80. 11354468 10.1080/10915810151115173

[10] Faiola B, Fuller ES, Wong VA, Recio L. Gene expression profile in bone marrow and hematopoietic stem cells in mice exposed to inhaled benzene. Mutat Res. 2004; 549:195–212. 15120971 10.1016/j.mrfmmm.2003.12.022

[11] Zhang Z, Wan J, Jin X, Jin T, Shen H, Lu D, et al Genetic Polymorphisms in XRCC1, APE1, ADPRT, XRCC2 and XRCC3 and Risk of Chronic Benzene Poisoning in a Chinese Occupational Population. Cancer Epidemiol Biomarkers Prev. 2005; 14: 2614–2619. 16284386 10.1158/1055-9965.EPI-05-0143

[12] Shen M, Lan Q, Zhang L, Chanock S, Li G, Vermeulen R, et al Polymorphisms in genes involved in DNA doublestrand break repair pathway and susceptibility to benzene-induced hematotoxicity. Carcinogenesis. 2006; 27: 2083–2089. 16728435 10.1093/carcin/bgl061

[13] Vidal AE, Boiteux S, Hickson ID, Radicella JP. XRCC1 coordinates the initial and late stages of DNA abasic site repair through protein-protein interactions. EMBO J. 2001; 20:6530–6539. 11707423 10.1093/emboj/20.22.6530 PMC125722

[14] Kubota Y, Nash RA, Klungland A, Schar P, Barnes D-E, Lindahl T. Reconstitution of DNA base excision-repair with purified human proteins: interaction between DNA polymerase h and the XRCC1 protein. EMBO J. 1996; 15: 6662–6670. 8978692 PMC452490

[15] Lu AL, Li X, Gu Y, Wright PM, Chang DY. Repair of oxidative DNA damage: mechanisms and functions. Cell Biochem Biophys. 2001; 35:141–170. 11892789 10.1385/CBB:35:2:141

[16] Nexø BA, Vogel U, Olsen A, Ketelsen T, Bukowy Z, Thomsen BL, et al A specific haplotype of single nucleotide polymorphisms on chromosome 19q13.2–3 encompassing the gene RAI is indicative of post-menopausal breast cancer before age 55. Carcinogenesis. 2003; 24: 899–904. 12771034 10.1093/carcin/bgg043

[17] Vogel U, Laros I, Jacobsen NR, Thomsen BL, Bak H, Olsen A, et al Two regions in chromosome 19q13.2–3 are associated with risk of lung cancer. Mutat Res. 2004; 546: 65–74. 14757194 10.1016/j.mrfmmm.2003.11.001

[18] Vogel U, Sorensen M, Hansen RD, Tjønneland A, Overvad K, Wallin H, et al Gene-environment interactions between smoking and a haplotype of RAI ASE-1 and ERCC1 polymorphisms among women in relation to risk of lung cancerin a population-based study. Cancer Lett. 2007; 247: 159–165. 16690207 10.1016/j.canlet.2006.04.001

[19] Yin J, Vogel U, Ma Y, Wang H, Wang C, Liang D, et al A specific diplotype defined by PPP1R13L rs1970764, CD3EAP rs967591 and ERCC1 rs11615 and lung cancer risk in a Chinese population. Lung Cancer. 2012; 76: 286–291. 10.1016/j.lungcan.2012.01.001 22335888

[20] Yin J, Rockenbauer E, Hedayati M, Jacobsen NR, Vogel U, Grossman L, et al Multiple single nucleotide polymorphisms on human chromosome 19q13.2–3 associate with risk of Basal cell carcinoma. Cancer Epidemiol Biomarkers Prev. 2002; 11:1449–1453. 12433725

[21] Skjelbred CF, Saebø M, Nexø BA, Wallin H, Hansteen IL, Vogel U, et al Effects of polymorphisms in ERCC1, ASE-1 and RAI on the risk of colorectal carcinomas and adenomas: a case control study. BMC Cancer. 2006; 6:175 16817948 10.1186/1471-2407-6-175 PMC1533843

[22] Nexø BA, Vogel U, Olsen A, Nyegaard M, Bukowy Z, Rockenbauer E, et al Linkage disequilibrium mapping of a breast cancer susceptibility locus near RAI/PPP1R13L/iASPP. BMC Med Genet. 2008; 9:56 10.1186/1471-2350-9-56 18588689 PMC2474586

[23] Jeon HS, Jin G, Kang HG, Choi YY, Lee WK, Choi JE, et al A functional variant at 19q13.3, rs967591G>A, is associated with shorter survival of early-stage lung cancer. Clin Cancer Res. 2013; 19: 4185–4195. 10.1158/1078-0432.CCR-12-2792 23775331

[24] Xiao S, Gao L, Liu Y, Yu T, Jin C, Pan L, et al Association of genetic polymorphisms in ERCC1 and ERCC2/XPD with risk of chronic benzene poisoning in a Chinese occupational population. Mutat Res. 2013; 751:52–58. 10.1016/j.mrgentox.2012.11.002 23147699

[25] Kim SY, Choi JK, Cho YH, Chung EJ, Paek D, Chung HW. Chromosomal aberrations in workers exposed too low levels of benzene: association with genetic polymorphisms. Pharmacogenetics. 2007; 14: 453–463. 10.1097/01.fpc.0000114751.08559.7b 15226677

[26] Maffei F, Hrelia P, Angelini S, Carbone F, Cantelli-Forti G, Barbieri A, et al Effects of environmental benzene: micronuclei frequencies and haematological values in traffic police working in an urban area. Mutat Res. 2009; 583: 1–11. 10.1016/j.mrgentox.2005.01.011 15866461

[27] Fracasso ME, Doria D, Bartolucci GB, Carrieri M, Lovreglio P, Ballini A, et al Low air levels of benzene: correlation between biomarkers of exposure and genotoxic effects. Toxicol Lett. 2010; 192: 22–28. 10.1016/j.toxlet.2009.04.028 19427373

[28] Leng S, Cheng J, Zhang L, Niu Y, Dai Y, Pan Z, et al The association of XRCC1 haplotypes and chromosomal damage levels in peripheral blood lymphocyte among coke-oven workers. Cancer Epidemiol Biomarkers Prev. 2005; 14:1295–1301. 15894689 10.1158/1055-9965.EPI-04-0690

[29] Stern MC, Umbach DM, van Gils CH, Lunn RM, Taylor JA. DNA repair gene XRCC1 polymorphisms, smoking, and bladder cancer risk. Cancer Epidemiol Biomarkers Prev. 2001; 10: 125–131. 11219769

[30] Chen S, Tang D, Xue K, Xu L, Ma G, Hsu Y, et al DNA repair gene XRCC1 and XPD polymorphisms and risk of lung cancer in a Chinese population. Carcinogenesis. 2002; 23: 1321–1325. 12151350 10.1093/carcin/23.8.1321

[31] Xing D, Qi J, Miao X, Lu W, Tan W, Lin D. Polymorphisms of DNA repair genes XRCC1and XPD and their associations with risk of esophageal squamous cell carcinoma in a Chinese population. Int J Cancer. 2002; 100: 600–605. 12124811 10.1002/ijc.10528

[32] Huang J, Ye F, Chen H, Lu W, Xie X. The nonsynonymous single nucleotide polymorphisms of DNA repair gene XRCC1 and susceptibility to the development of cervical carcinoma and high-risk human papillomavirus infection. Int J Gynecol Cancer. 2007; 17: 668–675. 17504380 10.1111/j.1525-1438.2007.00840.x

[33] Ding P, Yang Y, Cheng L, Zhang X, Cheng L, Li C, et al The relationship between seven common polymorphisms from five DNA repair genes and the risk for breast cancer in northern Chinese women. PLoS ONE. 2014; 9: e92083 10.1371/journal.pone.0092083 24642895 PMC3958445

[34] Jiang H, Wu D, Ma D, Lin G, Liang J, Jin J. Association between X-ray repair cross-complementation group 1 rs25487 polymorphism and pancreatic cancer. Tumor Biology. 2013; 34: 3417–3421. 10.1007/s13277-013-0914-9 23807675

[35] Chanvaivit S, Navasumrit P, Hunsonti P, Autrup H, Ruchirawat M. Exposure assessment of benzene in Thai workers, DNA-repair capacity and influence of genetic polymorphisms. Mutat Res. 2007; 626:79–87. 17095285 10.1016/j.mrgentox.2006.09.007

[36] Lee EB, Jeon HS, Yoo SS, Choi YY, Kang HG, Cho S, et al Polymorphisms in apoptosis-related genes and survival of patients with early-stage non-small-cell lung cancer. Ann Surg Oncol. 2010; 17:2608–2618. 10.1245/s10434-010-1082-4 20422457

[37] Chae YS, Kim JG, Kang BW, Lee SJ, Jeon HS, Park JS, et al PPP1R13L variant associated with prognosis for patients with rectal cancer. J Cancer Res Clin Oncol. 2013; 139:465–473. 10.1007/s00432-012-1346-4 23180017 PMC11824692

[38] Bergamaschi D, Samuels Y, O’Neil NJ, Trigiante G, Crook T, Hsieh JK, et al iASPP oncoprotein is a key inhibitor of p53 conserved from worm to human. Nat Genet. 2003; 33: 162–167. 12524540 10.1038/ng1070

[39] Vousden KH. Outcomes of p53 activation-spoilt for choice. J Cell Sci. 2006; 119: 5015–5020. 17158908 10.1242/jcs.03293

[40] Recio L, Bauer A, Faida B. Use of genetically mouse models to assess pathways of benzene-induced bone marrow cytotoxicity and genotoxicity. Chem Biol Interact. 2005; 153–154: 159–164. 15935812 10.1016/j.cbi.2005.03.020

