# Supplementary figures and images for: Genetic Polymorphisms in XRCC1, CD3EAP, PPP1R13L, XPB, XPC, and XPF and the Risk of Chronic Benzene Poisoning in a Chinese Occupational Population
Source: PLoS One. 2015 Dec 17;10(12):e0144458. doi: 10.1371/journal.pone.0144458 (PMC4683048; doi:10.1371/journal.pone.0144458)

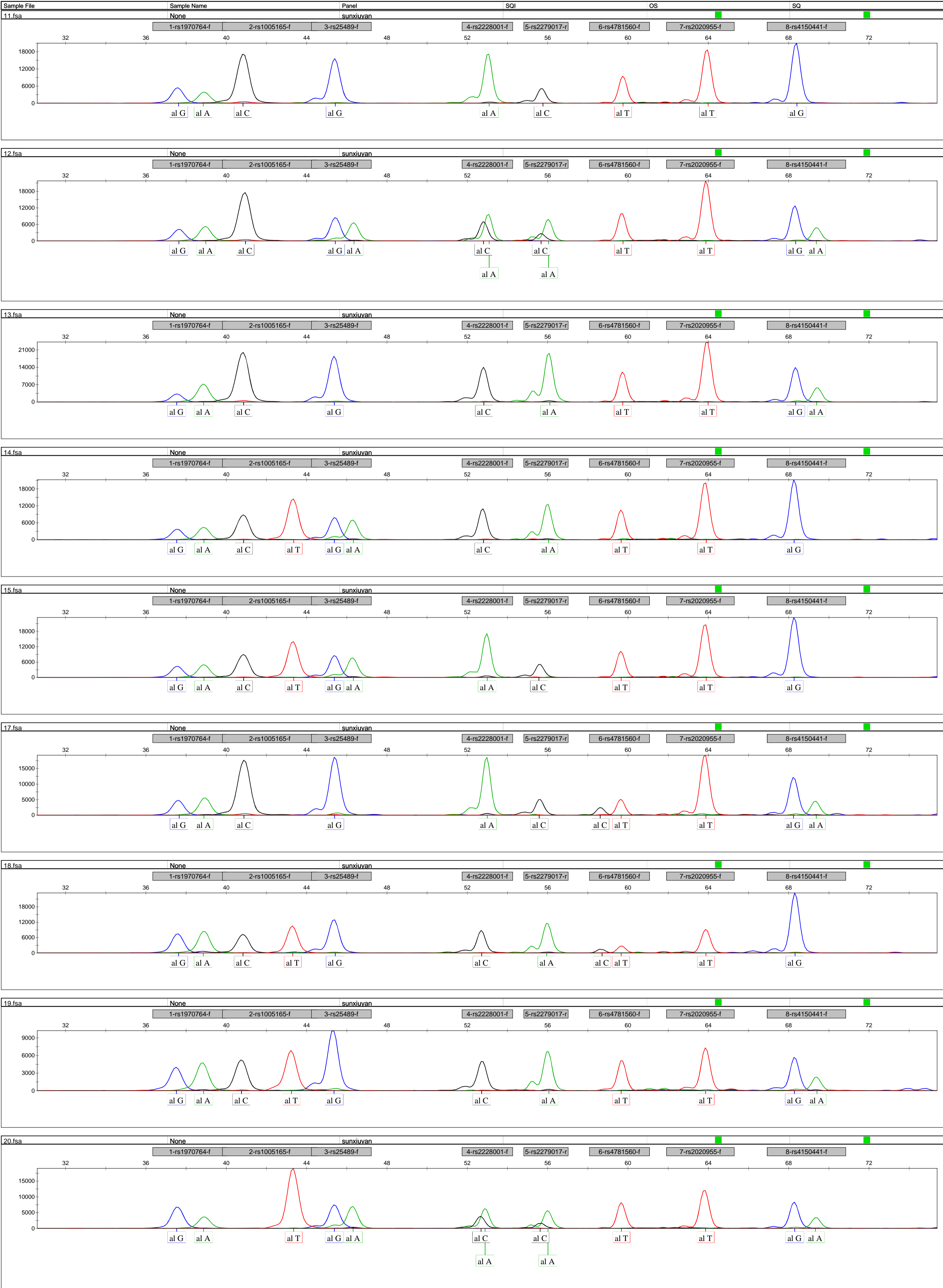

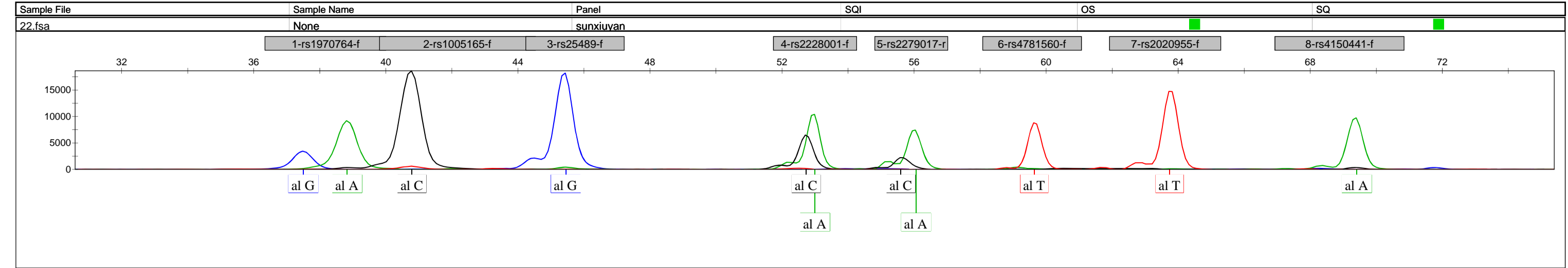

Supplement: S1 Fig — GeneMapper electropherograms of SNaPshot reactions. Plots of size (nt) versus relative fluorescence units (rfus) for 10 DNA samples exhibiting variations at the SNP sites (PPP1R13L rs1970764 and XPF rs2020955 were not chose for our data analysis at last). The x axis represents the size (bp) of the primer pair with the incorporated nucleotides, while the y axis corresponds to the relative fluorescent units of the peak. Each fluorescent dye corresponds to a different nucleotide: blue represents G, green represents A, red represents T, and black represents C. The orange peak represents the size standard. (PDF) [file pone.0144458.s001.pdf]
