# Supplementary material for: Genetic Polymorphisms in XRCC1, CD3EAP, PPP1R13L, XPB, XPC, and XPF and the Risk of Chronic Benzene Poisoning in a Chinese Occupational Population
Source: PLoS One. 2015 Dec 17;10(12):e0144458. doi: 10.1371/journal.pone.0144458 (PMC4683048; doi:10.1371/journal.pone.0144458)
Supplement: S1 Supporting Information — (PDF) [file pone.0144458.s002.pdf]

Applied Biosystems

High-Throughput SNP Genotyping Using the ABI PRISM® 3100 Genetic Analyzer with 22-cm Capillary Array

The ABI PRISM® 3100 Genetic Analyzer, used together with the new 22-cm capillary array and the ABI PRISM® SNaPshot® Multiplex Kit, is an effective solution for the rapid validation and screening of single-nucleotide polymorphisms (SNPs). With this combination, researchers can accurately multiplex up to 10 single-base primer-extension reactions per capillary on the 3100 system at an overall throughput of up to 15,360 SNP genotypes in 24 hours (twice the throughput of SNP genotyping when using the standard 36-cm array on the 3100 system).

SNP Analysis and the ABI PRISM SNaPshot Multiplex Kit

Many methods are currently available for characterizing single-nucleotide polymorphisms (SNPs). These methods include microarray scanning, real-time PCR analysis, and MALDI-TOF. Electrophoretic techniques, such as single-nucleotide primer extension (also called single-base extension), OLA analysis, and dideoxy sequencing are also used.

The SNaPshot Multiplex Kit uses single-base extension to provide a solution for SNP validation. The system's Multiplex Ready Reaction Mix enables robust multiplex SNP interrogation of PCR-generated templates. SNaPshot multiplexing can be accomplished by spatially representing multiple SNP products. This is achieved by tailing the 5' end of the unlabeled SNaPshot primers with varying lengths of noncomplementary oligonucleotide sequences that serve as a mobility modifier. During the reaction, the sequence-specific probe interrogates each locus and labels it at the 3' end using fluorescent dideoxynucleotide chemistry. The resulting products can then be separated electrophoretically in a single capillary and analyzed in the presence of a fifth-dye-labeled size standard.

ABI PRISM 3100 Genetic Analyzer: Complete System for Rapid, Cost-Effective SNP Analysis

- **ABI PRISM 3100 Genetic Analyzer 22-cm Array**
  Doubles the throughput of SNP analysis on the 3100 system without compromising precision, resolution, or multiplexing capability. The high-quality data is accurate and precise, yielding ± 0.5 bp standard deviation for up to 105 bp.
- **SNaPshot Multiplex Kit**
  Provides the necessary reagents to perform high-throughput SNP validation on an existing 3100 Genetic Analyzer.

  SNPs can be interrogated regardless of their position on the chromosome or separation from a neighboring SNP locus. User-defined unlabeled primers let researchers cost-effectively incorporate SNPs of interest.

  Applied Biosystems recommends limiting SNP marker size to between 20 and 105 bp. Loci less than 36 bp should be spaced a minimum of 6 bp apart, while loci greater than 36 bp should be spaced 4 bp apart. See the user bulletin "ABI PRISM 3100 22-cm Capillary Array for High Throughput Microsatellite and SNP Genotyping" for information on microsatellite analysis.
- **GeneScan™-120 LIZ™ Size Standard**
  Accurately sizes samples ranging from 20 to 110 nucleotides. Eliminates manual allele calling when used with GeneMapper v 2.0.

ABI PRISM 3100 Genetic Analyzer: SNP Genotyping Throughput per Array Length

Array Length	Polymer Type	Run Time	Runs/24 hours	Number of Capillaries	Number of Loci	Genotypes/24Array hours
22-cm*	POP-4™	15	96	16	10	15,360
36-cm	POP-4™	30	48	16	10	7,680

* The use of the 22-cm array is not recommended for HID/Forensics applications.

- **GeneMapper™ Software v 2.0**
  A Windows NT®/Windows 2000 OS-based software tool designed to provide high throughput and accurate allele calling for SNP- and microsatellite-based genotyping. Unique Process Component-Based Quality Values (PQVs) and specialized algorithms filter out problematic data and identify poor-quality data, reducing the need to examine allele calls. For SNP-based genotyping, electropherograms can be displayed with multiplexed markers and bin overlays to facilitate quick visualization of allele calls. GeneMapper Software v 2.0 can analyze over 4,000 genotypes within an hour—six times faster than Genotyper® Software v 3.7.

- **Matrix Standard Set DS-02**
  Ensures successful, rapid five-dye (dR10, dR6G, dTAMRA™, dROX™, and LIZ™) SNP analysis on the ABI PRISM 3100 Genetic Analyzer.

Conclusion

Used with the new 22-cm capillary array, the single-base extension technology of the ABI PRISM SNaPshot Multiplex Kit, and POP-4™ polymer—the ABI PRISM 3100 Genetic Analyzer is an ideal method for high-throughput SNP validation and screening. With the addition of GeneMapper Software v 2.0, specially designed to provide high throughput and accurate allele calling for SNP- and microsatellite-based genotyping, the 3100 system can automatically score over 4,000 genotypes per hour—with unparalleled ease and accuracy.

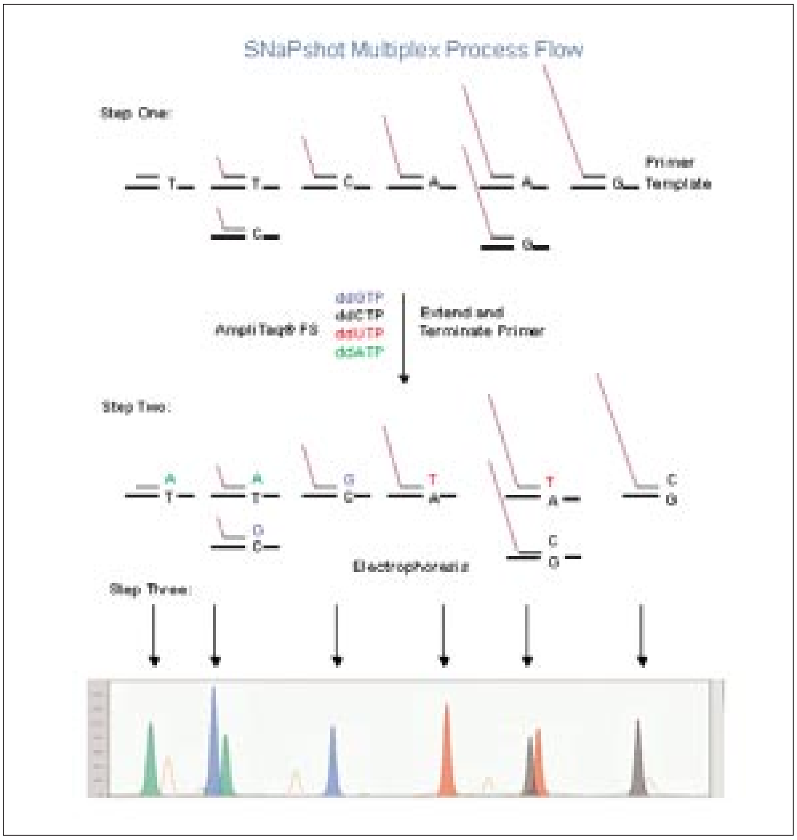

Figure 1. Step One: Perform PCR amplification and dilute PCR products.
Step Two: Design differing length tailed primers, perform Single Base Extension Reaction with SNaPshot Multiplex kit.
Step Three: Separate fragments and detect on Capillary Electrophoresis Platform. Opaque peaks show the single extension base products and clear peaks show the GS-120 size standards.

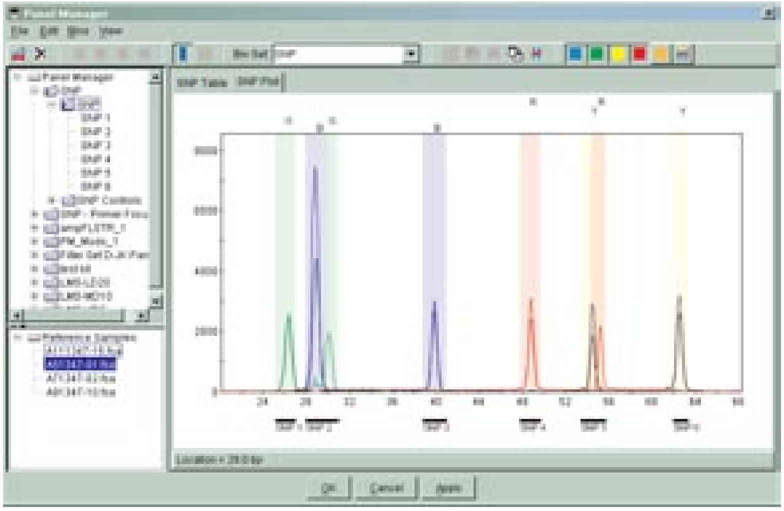

Figure 2. GeneMapper™ Software V 2.0 is capable of analyzing SNaPshot Multiplex Kit data. The view above displays a reference 6plex SNP sample with bins overlaid. Bin information for each loci can be defined manually by the user or by using the Auto Panel feature to optimize marker distribution.

SNaPshot Multiplex Kit 5-Dye Set

dRhodamine Terminator Set and Size Standard

ddNTP	Dye	Color
A	dR6G	Green
C	dTAMRA™	Yellow (black)
G	dR110	Blue
U (T)	dROX™	Red
Size standard	LIZ™	Orange

Ordering Information

Description	Comments	P/N
22-cm Capillary Array	For ABI PRISM 3100 Genetic Analyzer	4319898
POP-4™ Polymer	For ABI PRISM 3100 Genetic Analyzer	4316355

SNaPshot Multiplex Kit	10-Plex Quantity	P/N
100-reaction kit with protocol	1,000 genotypes	4323151
1,000-reaction kit with protocol	10,000 genotypes	4323154
5,000-reaction kit with protocol	50,000 genotypes	4323155

SNaPshot Multiplex Kit*		P/N
100-reaction kit*	1,000 genotypes	4323159
1,000-reaction kit*	10,000 genotypes	4323161
5,000-reaction kit*	50,000 genotypes	4323163

*Protocol not included

Size Standard (sold separately)	Comments	P/N
GeneScan™-120 LIZ™ Size Standard	800 capillary runs	4324287

Reagents and Software (sold separately)		P/N
HiDi Formamide		4311320
10X Genetic Analyzer Buffer with EDTA		402824
Matrix Standard Set DS-02	For ABI PRISM 3100 Genetic Analyzer	4323014
GeneMapper Software v 2.0	Initial license	4319372
GeneMapper Software v 2.0	Multiple licenses	4319374
GeneMapper Software v 2.0	Upgrade	4332537
GeneMapper v 2.0 with GeneScan 3.7 Software	For Macintosh®/Windows NT® system conversion	4327546

Worldwide Sales Offices

Applied Biosystems vast distribution and service network, composed of highly trained support and applications personnel, reaches into 150 countries on six continents. For international office locations, please call the division headquarters or refer to our Web site at **www.appliedbiosystems.com.**

Applera Corporation is committed to providing the world's leading technology and information for life scientists. Applera Corporation consists of the Applied Biosystems and Celera Genomics businesses.

Headquarters

850 Lincoln Centre Drive
Foster City, CA 94404 USA
Phone: 650.638.5800
Toll Free: 800.345.5224
Fax: 650.638.5884

For Research Use Only.
Not for use in diagnostic procedures.

The ABI PRISM® 3100 Genetic Analyzer includes patented technology licensed from Hitachi, Ltd. As part of a strategic partnership between Applied Biosystems and Hitachi, Ltd., as well as patented technology of Applied Biosystems.

AB (Design), Applera, dTAMRA, dROX, GeneMapper, Genotyper, LIZ , POP-4 are trademarks and ABI PRISM, Applied Biosystems, GeneScan are registered trademarks of Applera Corporation or its subsidiaries in the US and certain other countries.

Oracle is a registered trademark of Oracle Corporation.

Windows NT is a registered trademark of Microsoft Corporation.

©2002 Applied Biosystems. All rights reserved.
Information subject to change without notice.

Printed in the USA, 04/2002,
Publication 106AP06-01

Applied Biosystems
