# Supplementary material for: Genetic Polymorphisms in XRCC1, CD3EAP, PPP1R13L, XPB, XPC, and XPF and the Risk of Chronic Benzene Poisoning in a Chinese Occupational Population
Source: PLoS One. 2015 Dec 17;10(12):e0144458. doi: 10.1371/journal.pone.0144458 (PMC4683048; doi:10.1371/journal.pone.0144458)
Supplement: S1 Table — (DOCX) [file pone.0144458.s003.docx]

**S1 Table.** **Description of the missing data**

| SNPs | Cases  n*(％) | Controls  n*(％) | Smoking | | No Smoking | | Alcohol consumption | | No Alcohol consumption | | Male | | Female | | Exposure duration(y)≤12 | | Exposure duration(y)>12 | |
| --- | --- | --- | --- | --- | --- | --- | --- | --- | --- | --- | --- | --- | --- | --- | --- | --- | --- | --- |
|  |  |  | Cases  n*(％) | Controls  n*(％) | Cases  n*(％) | Controls  n*(％) | Cases  n*(％) | Controls  n*(％) | Cases  n*(％) | Controls  n*(％) | Cases  n*(％) | Controls  n*(％) | Cases  n*(％) | Controls  n*(％) | Cases  n*(％) | Controls  n*(％) | Cases  n*(％) | Controls  n*(％) |
| XRCC1 rs25487 | 1(0.98) | 2(0.98) | 0(0) | 0(0) | 1(1.12) | 2(1.03) | 0(0) | 0(0) | 1(1.16) | 2(1.11) | 0(0) | 1(2.38) | 1(1.23) | 1(0.62) | 1(1.61) | 1(0.76) | 0(0) | 1(1.36) |
| XRCC1 rs25489 | 1(0.98) | 4(1.96) | 0(0) | 0(0) | 1(1.12) | 4(2.07) | 0(0) | 0(0) | 1(1.16) | 4(2.22) | 1(4.76) | 1(2.38) | 0(0) | 3(1.85) | 1(1.61) | 3(2.29) | 0(0) | 1(1.36) |
| XRCC1 rs1799782 | 0(0) | 3(1.47) | 0(0) | 0(0) | 0(0) | 3(1.55) | 0(0) | 0(0) | 0(0) | 3(1.67) | 0(0) | 1(2.38) | 0(0) | 2(1.23) | 0(0) | 2(1.52) | 0(0) | 1(1.36) |
| PPP1R13Lrs1005165 | 1(0.98) | 4(1.96) | 0(0) | 0(0) | 1(1.12) | 4(2.07) | 0(0) | 0(0) | 1(1.16) | 4(2.22) | 1(4.76) | 1(2.38) | 0(0) | 3(1.85) | 1(1.61) | 3(2.29) | 0(0) | 1(1.36) |
| CD3EAP rs967591 | 3(2.94) | 2(0.98) | 0(0) | 0(0) | 3(3.37) | 2(1.03) | 0(0) | 0(0) | 3(3.48) | 2(1.11) | 0(0) | 1(2.38) | 3(3.7) | 1(0.62) | 2(3.22) | 1(0.76) | 1(2.50) | 1(1.36) |
| XPB rs4150441 | 1(0.98) | 4(1.96) | 0(0) | 0(0) | 1(1.12) | 4(2.07) | 0(0) | 0(0) | 1(1.16) | 4(2.22) | 1(4.76) | 1(2.38) | 0(0) | 3(1.85) | 1(1.61) | 3(2.29) | 0(0) | 1(1.36) |
| XPC rs2279017 | 1(0.98) | 4(1.96) | 0(0) | 0(0) | 1(1.12) | 4(2.07) | 0(0) | 0(0) | 1(1.16) | 4(2.22) | 1(4.76) | 1(2.38) | 0(0) | 3(1.85) | 1(1.61) | 3(2.29) | 0(0) | 1(1.36) |
| XPC rs2228001 | 1(0.98) | 4(1.96) | 0(0) | 0(0) | 1(1.12) | 4(2.07) | 0(0) | 0(0) | 1(1.16) | 4(2.22) | 1(4.76) | 1(2.38) | 0(0) | 3(1.85) | 1(1.61) | 3(2.29) | 0(0) | 1(1.36) |
| XPF rs4781560 | 1(0.98) | 4(1.96) | 0(0) | 0(0) | 1(1.12) | 4(2.07) | 0(0) | 0(0) | 1(1.16) | 4(2.22) | 1(4.76) | 1(2.38) | 0(0) | 3(1.85) | 1(1.61) | 3(2.29) | 0(0) | 1(1.36) |

*number of missing value
